# SV40 T-antigen uses a DNA shearing mechanism to initiate origin unwinding

**DOI:** 10.1073/pnas.2216240119

**Published:** 2022-11-28

**Authors:** Lance D. Langston, Zuanning Yuan, Roxana Georgescu, Huilin Li, Michael E. O’Donnell

**Affiliations:** ^a^DNA Replication Laboratory, The Rockefeller University, New York, NY 10065; ^b^HHMI, Chevy Chase, MD 20815; ^c^The Rockefeller University, New York, NY 10065; ^d^Department of Structural Biology Van Andel Institute, Grand Rapids, MI 49503

**Keywords:** replication origins, SV40 T-Antigen helicase, DNA shearing mechanism, origin activation, origin unwinding

## Abstract

Duplication of DNA genomes requires unwinding of the double-strand (ds) DNA so that each single strand (ss) can be copied by a DNA polymerase. The genomes of eukaryotic cells are unwound by two ring-shaped hexameric helicases that initially encircle dsDNA but transition to ssDNA for function as replicative helicases. How the duplex is initially unwound, and the role of the two helicases in this process, is poorly understood. We recently described an initiation mechanism for eukaryotes in which the two helicases are directed inward toward one another and shear the duplex open by pulling on opposite strands of the duplex while encircling dsDNA [L. D. Langston, M. E. O'Donnell, *eLife* **8**, e46515 (2019)]. Two head-to-head T-Antigen helicases are long known to be loaded at the SV40 origin. We show here that T-Antigen tracks head (N-tier) first on ssDNA, opposite the direction proposed for decades. We also find that SV40 T-Antigen tracks directionally while encircling dsDNA and mainly tracks on one strand of the duplex in the same orientation as during ssDNA translocation. Further, two inward directed T-Antigen helicases on dsDNA are able to melt a 150-bp duplex. These findings explain the “rabbit ear” DNA loops observed at the SV40 origin by electron microscopy and reconfigure how the DNA loops emerge from the double hexamer relative to earlier models. Thus, the mechanism of DNA shearing by two opposing helicases is conserved in a eukaryotic viral helicase and may be widely used to initiate origin unwinding of dsDNA genomes.

## Significance

The initiation of replication of duplex DNA genomes requires that the duplex be opened up for helicase unwinding and to enable assembly of replisome machines that act at DNA replication forks. Cellular and many viral DNA genomes utilize two hexameric helicases that encircle duplex DNA. How two helicases that encircle duplex DNA are able to unwind the duplex is unknown, but this is required for assembly of the DNA replication machinery. This study reveals a DNA shearing mechanism of duplex unwinding by head-to-head SV40 T-Antigen helicases while they encircle duplex DNA. We propose that the mechanism generalizes to other systems including archaeal and eukaryotic origin initiation.

Decades of studies of Simian Virus 40 (SV40), with its double-stranded closed circular DNA genome and its key gene product, the large T-Antigen helicase (T-Ag), provided fundamental early insights into eukaryotic DNA replication (1, 2). Replication of the viral genome relies almost completely on host proteins, with T-Ag being the only essential viral gene product (3–5). This allowed for the identification of numerous host cell factors whose roles in DNA replication are now well known including DNA polymerases α and δ, RFC, PCNA, and RPA (1).

**Fig. 1.**
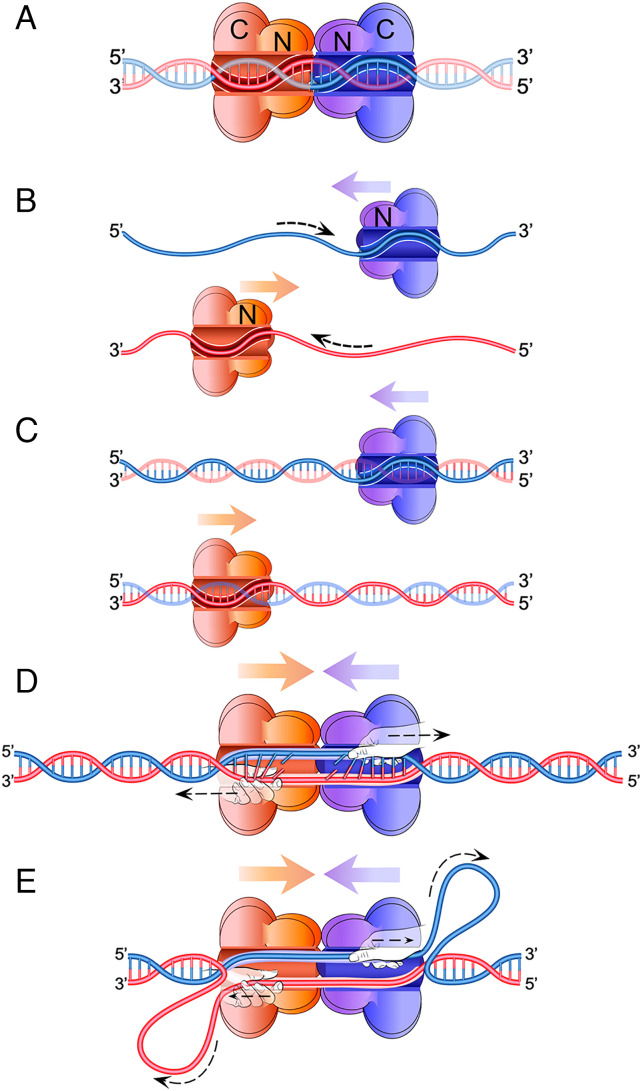
Essential features of the DNA shearing model of origin unwinding. (*A*) Two hexameric helicases are loaded head-to-head (N-face to N-face) surrounding dsDNA at origins of replication. The cutaway view shows the origin dsDNA inside the central channels of the two helicases. (*B*) The helicases are known to translocate 3′-5′ on ssDNA, and we show in this report that T-Ag is oriented N-first for translocation on ssDNA, as we found earlier for the CMG eukaryotic helicase (18). The colored arrows show the movement of the helicase with respect to the DNA, and the dotted black arrows show the movement of the DNA with respect to the helicase. (*C*) Even though the hexameric helicase encircles dsDNA, it makes primary contact with the same 3′–5′ tracking strand as in (*B*) for ssDNA. (*D*) Because the helicases are both surrounding dsDNA, they block each other’s N-first forward movement along the DNA, and we propose that the C-terminal AAA+ motors of each helicase (shown as hands pulling on the ssDNA) continue to track on their respective strands, shearing the duplex DNA apart between the two motors. (*E*) Continued tracking on their respective DNA strands leads to continued DNA shearing, with the unwound ssDNA spooling behind each helicase as growing ssDNA loops, while the distal ends of the duplex DNA are drawn toward the center. Image credit: Nina Yao.

To initiate viral genome duplication, two T-Ag hexamers have been shown to bind to a specific sequence in the viral genome known as the origin (6, 7). Monomers/dimers of T-Ag assemble from solution into ring-shaped homohexamers that topologically surround duplex DNA at two distinct positions in the origin to form a double hexamer (8–10). Like all other replicative helicases studied to date, the two hexamers load in opposite orientations on the DNA such that they are head-to-head (N-face to N-face) as illustrated in Fig. 1*A* (11–14). Assembly of the two hexamers leads to adenosine ribonucleoside triphosphate (ATP) hydrolysis-dependent unwinding of the origin duplex DNA. Because the two hexamers are assembled around duplex DNA, they cannot pass one another; so it has long been assumed that they travel away from one another C-first to initiate replication, with the C-terminal motor domains facing the replication fork (15–17).

One distinctive feature of the early stages of SV40 origin unwinding is that the two hexamers often remain juxtaposed to one another in electron micrographs (19). Thus, rather than the two rings moving separately along the DNA, the duplex DNA is reeled into the double hexamer, and ssDNA is extruded to form “rabbit ears” adjacent to it (19). This action has routinely been interpreted to indicate that ssDNA is being extruded at the interface between the two hexamers, but no conclusive data have ever been presented to support this assumption as the early EM studies were of far too low resolution to determine where DNA loops were extruded along the double-hexamer axis. The goal of the present work was to investigate the mechanism by which duplex DNA is initially unwound at the SV40 origin.

Like the replicative helicases of eukaryotes and some of their dsDNA viruses, T-Ag is a member of the AAA^+^ family, which share a highly conserved two-domain ATPase module that binds ATP (20, 21). T-Ag is further classified as a member of Superfamily III (SF3) helicases along with the well-studied bovine papillomavirus (BPV) E1 replicative helicase (22–24). The eukaryotic replicative helicase is the 11-subunit Cdc45/Mcm2-7/GINS (CMG) assembly*,* having an SF6 AAA+ helicase core composed of the six-subunit Mcm2-7 motor along with Cdc45 and the four-subunit GINS complex (25, 26) that scaffold other replisome factors (27). In common with all other SF3/SF6 helicases, CMG and T-Ag translocate 3’-5’ on ssDNA (28–30).

Like T-Ag, CMG is initially loaded at origins as two head-to-head (N-to-N) Mcm2-7 rings that topologically surround duplex DNA as shown in Fig. 1*A* (27, 31, 32). On its own, the MCM double hexamer has contact to both strands of dsDNA and is functionally inert (33, 34), but during the origin licensing process, the MCMs associate with the Cdc45 and GINS complex to become active CMG (Cdc45, Mcm2-7, GINS) helicases (25, 26). The transition from initial origin unwinding to active CMG translocation also requires the DNA binding protein Mcm10, though its exact function in this process is still unknown (35–39).

Although the CMG motors surround both strands of the DNA at an origin, they make primary contacts with only one strand of the duplex, the same strand that is used for ssDNA translocation as depicted in Fig. 1 *B* and *C* (40). We recently showed that the CMG ring moves with its N-terminal domains facing forward in the direction of translocation (Fig. 1*B*), opposite to what had previously been assumed (18). Thus, when two CMGs are loaded in a head-to-head (N-to-N) orientation around dsDNA at an origin, they are poised to translocate toward one another while contacting opposite strands of the duplex. Because the two CMG rings surround duplex DNA, they cannot pass one another. Thus, the dsDNA is likely reeled into the double-hexameric CMG with each ring’s motors pulling on opposite strands, ripping or shearing the strands apart, as our model studies indicated (Fig. 1*D*). Specifically, the base pairs linking the two strands are disrupted in the duplex DNA between the opposing motors, and then ssDNA is spooled into or behind each ring as the motors continue to actively track on their respective strands (Fig. 1*E*). In agreement with other cellular and biochemical studies, this shearing action of CMG also requires the Mcm10 protein (40).

Given the similarities between CMG and T-Ag, which are both constructed from AAA+ domains and loaded as double hexamers around dsDNA in a head-to-head (N-to-N) orientation at their respective origins, we considered whether the mechanism observed for initial origin melting and unwinding by CMG might also operate in the classic SV40 system for origin unwinding by T-Ag. To test this proposal, we investigated three key questions about T-Ag that had not, to our knowledge, been previously studied. First, what is the orientation of T-Ag when translocating on DNA, i.e., does it move with its N-tier forward during ssDNA translocation as in Fig. 1*B* or do the two hexamers move away from one another C-first as previously assumed? Second, does T-Ag track directionally while encircling dsDNA? Third, does it track on only one strand, not two, while encircling duplex DNA?

These are the three fundamental questions that need to be addressed to support a DNA shearing mechanism of duplex DNA unwinding at the SV40 origin by T-Ag, as shown in Fig. 1. We address these and find that all three criteria apply to T-Ag. We then experimentally demonstrate that two T-Ag helicases, when loaded to encircle dsDNA at opposite ends of one DNA molecule in head-to-head orientation, can indeed rip/shear over 100 bp of dsDNA apart to ssDNAs as previously observed for two head-to-head CMG helicases that encircle dsDNA (40). Considering that this DNA shearing mechanism of duplex unwinding occurs in both eukaryotes and the SV40 virus, the mechanism first described for yeast CMG might be widely used not only by cellular and viral replication initiator proteins in eukaryotes but possibly in other domains as well.

## Results

### The N-Terminal Ends of the T-Ag Hexamer (i.e. N-Tier) Faces toward the 5′ End of ssDNA.

**Fig. 2.**
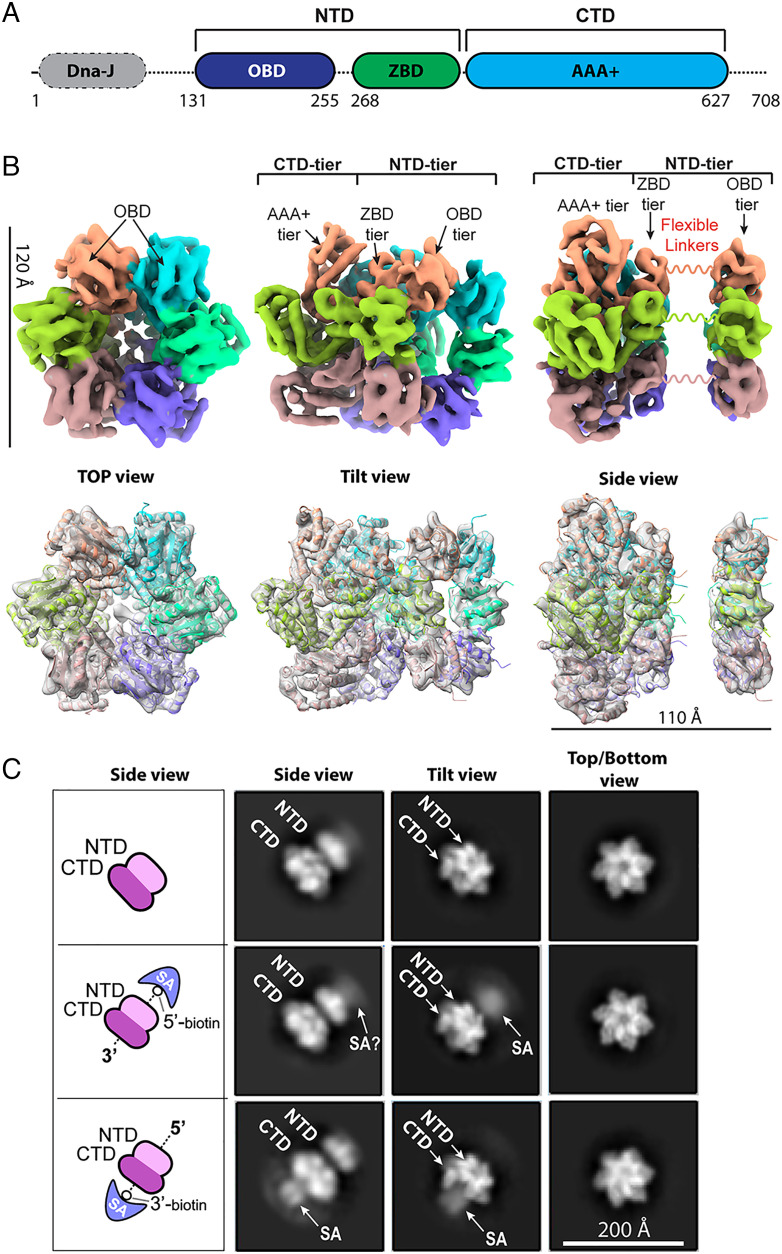
The N-tier of T-Ag faces toward the 5′ end of ssDNA. T-Ag containing amino acid residues 131–627 was incubated with AMP-PNP, either in the absence of DNA or in the presence of two separate 20-mer oligonucleotides, one of which is biotinylated at the 5′ end and the other at the 3′ end, as indicated by illustrations in panel *C* of the figure. Streptavidin (SA) was added after adding the T-Ag^131–627^. (*A*) Domain architecture of T-Ag. (*B*) Cryo-EM 3D map of T-Ag hexamer (*Upper* panel) and rigid-body docking with partial atomic models (*Lower* row; PDB ID 1SVM for AAA+ and ZBD, and 2FUF for OBD) shown in three orthogonal views. Subunits are individually colored. The OBD and ZBD domains, comprising the N-tier of T-Ag are connected to the C-tier region by a 14 residue disordered region, as indicated by squiggly lines between the OBD and the ZBD-AAA+ domains. (*C*) 2D class averages are shown for the side view, the tilted view, and the top view for each set of reactions. Comparison of the samples with biotin-SA-DNA to those without DNA indicates the presence of additional density representing SA at either the N- or C-tier of T-Ag depending on whether the oligo was biotinylated at the 3′ or 5′ end. The question mark in one panel indicates a density too light to definitively assign to SA.

T-Ag contains four domains (Fig. 2*A*): an N-terminal DnaJ homology domain (J domain), an origin binding domain (OBD), a Zn^2+^ binding domain (ZBD), and the C-terminal AAA+ domain (41). A truncated T-Ag missing both DnaJ and OBD domains was previously shown to assemble a hexamer (42). The isolated OBD has a Rossmann-like fold with a five-stranded β-sheet flanked at each side by two α-helices; but the isolated domain assembled a left-handed spiral in the crystal with 6_5_ helical symmetry (43). Interestingly, when OBD is included in the construct excluding only the N-terminal DnaJ domain, T-Ag^131–627^ assembled a dimer to bind the origin dsDNA (44). Therefore, it is unclear how the OBD arranges in a full-length T-Ag hexamer. We determined a cryo-EM map of the T-Ag^131–627^ hexamer at 5.6-Å resolution (Fig. 2*B* and *SI Appendix*, Fig. S1). By docking the available crystal structures of smaller fragments, we found this near full-length T-Ag construct assembled a hexamer with the OBD forming a ring on top. The OBD ring is separated by 20 Å from, and appears to be floating above the main body, consistent with the presence of an extended and likely disordered linker (14 amino acids) between the two regions. This “Flexible Linker” region is not sufficiently ordered to obtain 3D structure and appears as a space between the OB domain and the remainder of the T-Ag. This is noted in Fig. 2*B*. The medium-resolution EM map helps to recognize features and establish orientation in the 2D class images as described below.

Despite extensive biochemical and structural studies, to date there are no structures of T-Ag that clearly resolve the orientation of the hexamer on ssDNA, its substrate during replication fork movement. Given that the two T-Ag hexamers at the SV40 origin are loaded with their 6 NTDs (referred to here as the “N-tier”) facing one another (11–13), the direction of translocation as a helicase on ssDNA determines whether they move away from one another or must pass one another when they initiate bidirectional replication of the SV40 genome (15). Even if the two T-Ag hexamers remain adjacent to one another as an apparent double-hexamer “factory” during the early stages of replication as previously observed (19), the orientation of T-Ag on the DNA during translocation critically determines the mechanism by which ssDNA is produced, as dsDNA is pulled into the paired hexamers. This will be addressed in detail in the *Results* and *Discussion*.

To determine the orientation of T-Ag on ssDNA, we used the same truncated form of T-Ag (T-Ag^131–627^) lacking the N-terminal DnaJ-homology domain as well as the extreme C-terminal host-range determination region, both of which are predicted to be disordered (11). Previous structural studies have shown that full-length T-Ag displays a great deal of conformational flexibility, particularly when the DnaJ-homology domain is present (13), while T-Ag^131–627^ yields high-resolution structures and retains robust helicase activity (44). We designed 20-mer ssDNA “pointers” with SA bound to biotin at either the 3′ or 5′ end of a 20-mer dT ssDNA oligo. We incubated T-Ag^131–627^ with the SA-bound 20-mer ssDNA pointers in separate reactions with AMP-PNP/Mg^2+^ and examined the protein-DNA complexes by cryo EM (Fig. 2*C*).

As shown in Fig. 2*C*, when the 20-mer ssDNA is biotinylated at the 5′ end, additional density attributable to SA is observed at the position of the 6 NTDs of T-antigen, which we refer to here as the N-tier of the T-Ag hexamer. This additional density at the N-tier of T-Ag is not seen in the absence of DNA. In contrast, when the 20-mer ssDNA is biotinylated at the 3’ end, additional SA density is observed adjacent to the C-tier of T-Ag in the presence of DNA (the 6 CTDs of the T-antigen hexamer subunits are referred to here as the C-tier of the T-Ag hexamer). Together, these results reveal the orientation of T-Ag on ssDNA for the first time. Specifically, they show that T-Ag is positioned N-first for 3′–5′ movement along DNA as illustrated in Fig. 1*B*. In support of this finding, we note that the yeast CMG, human CMG, fly CMG, archaeal Mcm homohexamer, and BPV E1 helicase all translocate 3′–5′ on ssDNA with their N-tier facing the 5’ end of the DNA (18, 28, 45–49). Thus, an N-first direction for 3′–5′ translocation appears to be widely conserved among SF3 and SF6 AAA+ replicative helicases. The N-first orientation of T-Ag on ssDNA is also consistent with the previously observed movement of the DNA binding β-hairpin in the AAA+ module of T-Ag, when ATP-bound and nucleotide-free states are compared in the absence of DNA (42).

### SV40 T-Ag Tracks Actively and Directionally while Surrounding Duplex DNA.

**Fig. 3.**
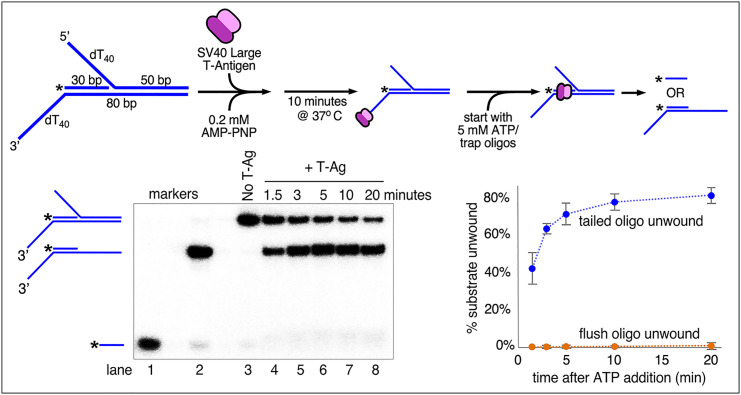
SV40 T-Antigen tracks directionally while encircling duplex DNA. The design of the substrate is shown in the schematic at *Top*, *Left*. The radiolabeled oligo in the substrate is indicated by an asterisk at the 5′ end. Wild-type T-Ag (40 nM as hexamer) is preincubated with the substrate (0.5 nM) for 10 min in the presence of 0.2 mM AMP-PNP before starting the reaction by addition of 5 mM ATP along with trap oligos to prevent reannealing of either the flush- or tailed-duplex oligo after unwinding (see *Materials and Methods* and *SI Appendix* for details). Lanes 1 and 2 in the native PAGE gel (*Bottom Left*) show the migration of the two potential products of unwinding. Lane 3 shows the unreacted substrate and lanes 4–8 show the time course of unwinding by T-Ag. The plot at the bottom right shows the time course of unwinding of the tailed duplex (blue circles) and flush duplex (orange circles). Values in the plot are the average of three independent experiments, and the error bars show the SD.

To determine if SV40 T-Ag can actively translocate while surrounding duplex DNA, we employed a strategy previously used for other hexameric helicases including *Escherichia coli* DnaB, archaeal MCM, and eukaryotic CMG, where the helicase must first traverse a flush (i.e., untailed) duplex region in order to unwind a downstream 5′-tailed duplex (50–53). T-Ag has previously been shown to translocate 3′–5′ on ssDNA (29, 30), so we used a substrate with a 3′ dT_40_ tail to help load T-Ag followed by a 30-bp flush duplex DNA section directly adjacent to a downstream 5′-tailed 50-bp duplex section (Fig. 3, schematic at *Top Left*). The current view is that T-Ag does not unwind a flush duplex well and would likely pass over the flush duplex. Thus, in order to unwind the 5′-tailed duplex, T-Ag must actively translocate over the flush duplex and unwind the 5′-tailed duplex, and this is what we observe in Fig. 3. The flush duplex oligo was radiolabeled at its 5′ end, and it was not unwound because if it were displaced, it would migrate in the gel much more quickly than when it is annealed to the tailed substrate (Fig. 3, lane 1). If the flush duplex is not unwound but the downstream 5′*-*tailed duplex is unwound, the substrate migrates at an intermediate position (Fig. 3, lane 2). As shown in the time course in Fig. 3, lanes 4–8, essentially none of the labeled flush duplex is unwound, but the downstream tailed duplex is rapidly and progressively unwound. The experiments of Fig. 3 contain traps for both the flush and tailed duplexes to prevent reannealing of any unwound duplex. From the experiment in Fig. 3, we conclude that T-Ag is capable of surrounding duplex DNA without unwinding it while translocating with sufficient shearing capability while on the duplex to initiate unwinding of the downstream 5′-tailed oligo.

### SV40 T-Ag Tracks on One Strand while Encircling Duplex DNA.

Next, we wanted to determine whether T-Ag contacts one or both DNA strands during duplex translocation. To do so, we used a similar substrate to that in Fig. 3 but with a longer 40-bp flush duplex section to accommodate a stretch of 20 bases connected by methylphosphonate (MeP) linkages on the DNA phosphate backbone of one DNA strand or the other. Unlike typical phosphodiester linkages that are negatively charged at physiological pH, MeP linkages are not charged. Many DNA translocases, including T-Ag, bind to the negatively charged phosphodiester backbone (22, 54, 55), and previous studies with T-Ag indicated that contacts with the negatively charged DNA backbone are more important for DNA unwinding than contacts with the DNA bases (56).

**Fig. 4.**
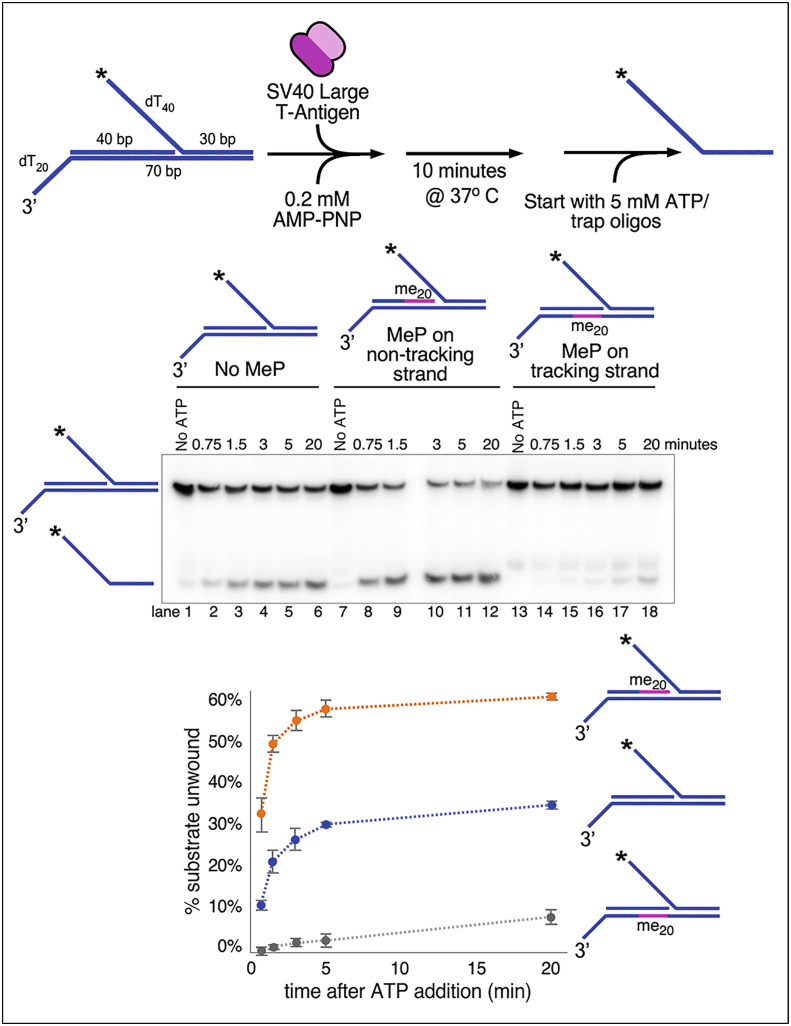
T-Ag mainly contacts one strand of dsDNA while moving directionally to unwind a downstream 5’ tailed DNA strand. The design of the substrate is shown in the reaction scheme at *Top, Left*. The radiolabeled oligo in the substrate is indicated by an asterisk at the 5’ end. As shown in pink on the schematics above the gel, the substrate contains 20 charge-neutralizing MeP linkages on the phosphate backbone of the tracking strand, (lanes 13–18), the nontracking strand (lanes 7–12) or neither strand (lanes 1–6). T-Ag (5 nM as hexamer) is preincubated with the substrate (0.5 nM) for 10 min in the presence of 0.2 nM AMP-PNP before starting the reaction by the addition of 5 mM ATP along with unlabeled trap DNA. The plot at the bottom shows the time course of unwinding of the substrate with no MePs (blue circles) or 20 MeP linkages on the tracking (gray circles) or nontracking (orange circles) strand. Values in the plot are the average of three independent experiments, and the error bars show the SD.

To examine the effect of removing charge in either of the DNA backbones during duplex DNA translocation, we used helicase substrates with a flush duplex region of 40 bp, where the 20 bases adjacent to the forked junction are connected by neutral MeP linkages on one strand or the other (Fig. 4). In these experiments, the 5′-tailed duplex was radiolabeled in order to observe whether the ability of T-Ag to unwind the 5′-tailed duplex is affected by neutralization of negative charges on the phosphate backbone of either strand of the flush duplex. We added ATP to start the reaction along with trap DNA to prevent reannealing of any unwound products.

The results of this experiment are shown in Fig. 4. When the MeP linkages were on the 3′–5′ strand in the direction of unwinding (i.e., the “tracking strand”, lanes 13–18 in Fig. 4), unwinding was greatly reduced compared to the substrate with no MePs (lanes 1–6, Fig. 4). This result strongly supports the idea that T-Ag interacts with the negatively charged phosphate backbone of the tracking strand during duplex DNA translocation, similar to what was observed for CMG tracking on dsDNA (40, 57). Somewhat surprisingly, the presence of MePs on the nontracking strand increased unwinding twofold compared with the substrate with no MePs (Fig. 4, compare lanes 7–12 to lanes 1–6). This result suggests that the negatively charged phosphate backbone of the nontracking strand has some contact with T-Ag that inhibits dsDNA translocation, and this inhibition is released when the DNA charge on the nontracking strand is neutralized. The physiological consequences of these possible secondary interactions are interesting and warrant further study.

Having established that T-Ag hexamers track directionally while encircling duplex DNA through contacts with mainly one strand of the duplex and with the N-tier forward in the direction of helicase movement, this implies that the two T-Ag hexamers loaded at the SV40 origin are running into each other and must pass one another to initiate bidirectional DNA replication. It also suggests that T-Ag might use a mechanism to initiate melting of the duplex DNA at the origin similar to the one recently proposed for yeast CMG where the motors of each ring track on opposite strands of the duplex, but because they initially encircle duplex DNA, they cannot pass one another (40). As the two opposing hexameric motors continue to pull on their respective tracking strands, the base pairs connecting the two strands between the motors are broken, and the duplex DNA is sheared apart (Fig. 1 *D*–*E*).

It is well known that T-Ag is capable of initiating duplex melting at the SV40 origin, and numerous mechanisms have been proposed for how this occurs, but most of these mechanisms involved extrusion of ssDNA through a proposed side-channel in the ring or direct melting of duplex DNA in conjunction with hexamer assembly around ssDNA. Furthermore, most of the models were based on the rings moving with the C-tier forward in the direction of movement. We propose instead that the two rings shear the DNA by tracking N-first toward one another on opposite strands while encircling duplex DNA with main connections to their respective opposite tracking strands and having sufficient pulling capability to rip the two strands apart.

### Two Oppositely Facing T-Ag Hxamers can Unwind Nonorigin Duplex DNA.

**Fig. 5.**
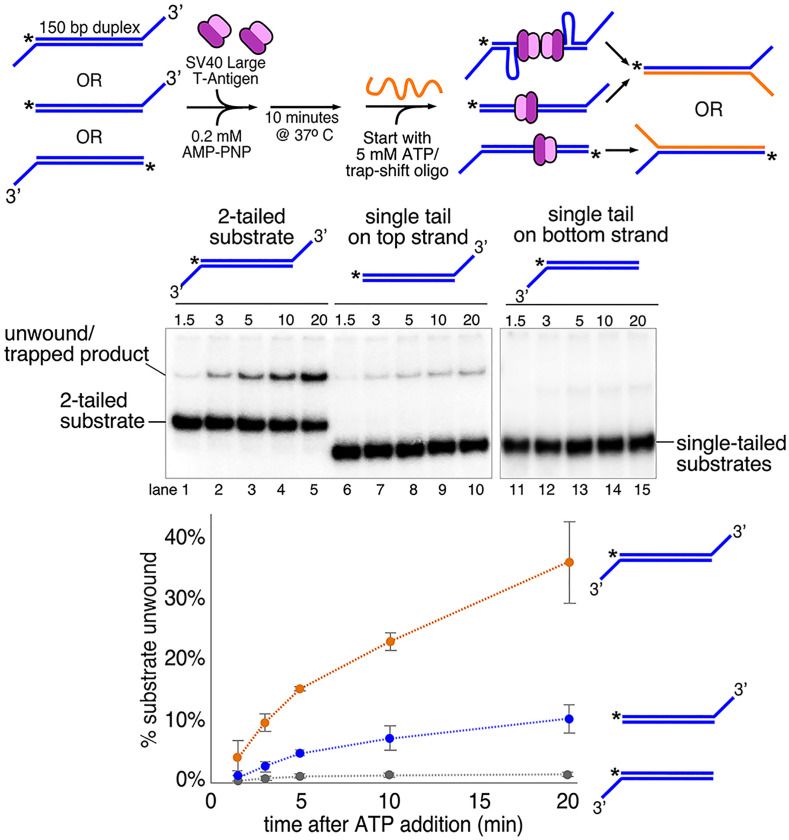
Two oppositely facing T-Ag hexamers can unwind duplex DNA. Loading of two T-Ag hexamers in opposing orientations leads to unwinding of nonorigin duplex DNA. A 150-bp duplex DNA with no SV40 origin sequences was assembled containing 3′ tails at one end or the other or at both ends. The radiolabeled oligo in the substrate is indicated by an asterisk at the 5’ end. T-Ag (40 nM as hexamer) is preincubated with the substrate (0.5 nM) for 10 min in the presence of 0.2 nM AMP-PNP before starting the reaction by the addition of 5 mM ATP along with trap oligos (shown in orange in the schematic at *Top*) that anneal to the unwound radiolabeled strand and shift it to a slower migrating position in the gel (see *SI Appendix* for details). The plot at the bottom shows the time course of unwinding of the substrate with two 3′ tails (orange circles) or a single 3′ tail on the top or bottom strand (blue and gray circles, respectively). Values in the plot are the average of three independent experiments, and the error bars show the SD.

To test the proposal that head-to-head T-Ag helicases can unwind dsDNA, we used a substrate that allows T-Ag to load onto duplex DNA without requiring specific SV40 origin sequences. A 150-bp duplex was assembled with 3′-dT_40_ tails at each end of the duplex to allow loading of a T-Ag hexamer at each end (Fig. 5). Addition of 3′ tails to one end or the other allows a small amount of unwinding (Fig. 5, lanes 6–10 and 11–15), likely by thermal fraying as previously observed for T-Ag (58). Only in the presence of both 3′ tails is robust unwinding observed (Fig. 5, lanes 1–5), consistent with a requirement to load two oppositely facing head-to-head (NTD-tier-to-NTD-tier) T-Ag hexamers that meet on the duplex and shear the duplex DNA apart while encircling dsDNA (Fig. 1*E*).

## Discussion

### Orientation of T-Antigen during DNA Unwinding.

T-Antigen has long been thought to move CTD-tier-first on ssDNA, possibly because the head-to-head (N-to-N) orientation at an origin would seem to make it impossible for the T-Antigen hexamers to move, since each hexamer blocks the other from moving in the NTD-tier-first direction. However, the orientation of T-Antigen while unwinding DNA has been questioned and has not been empirically determined (59, 60). The N-terminal domain (NTD) tier-first orientation of T-Ag, determined herein by cryo-EM (Fig. 2), is opposite the long-held view of CTD-tier-first movement, and it has significant implications for the origin unwinding mechanism of T-Ag and invalidates previous models almost all of which were based on C-terminal domain (CTD) tier-first movement and the “iris hypothesis” (e.g., refs. 16 and 17).

**Fig. 6.**
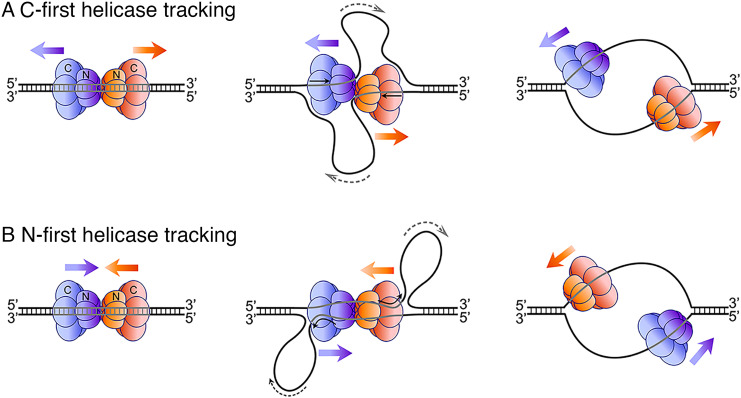
Contrast between C-first and N-first T-Ag helicase tracking at origins. The first illustrations of both panels (*A* and *B*) show the head-to-head (N-to-N) double hexamer of T-Ag, but the arrows denote whether they: (*A*) move C-first in opposite directions on DNA, or (*B*) move N-first into one another in which case they must pass each another on ssDNA. For two T-Ag hexamers that track C-first and simply depart in opposite directions, the observation of “rabbit ear” DNA loops suggests that the two T-Ag must remain connected together and draw DNA toward them to form the ssDNA loops. However, N-first tracking does not require long-lasting direct connections among T-Ag hexamers, and it pits the two T-Ag against one another to shear DNA and form “rabbit ear” ssDNA loops. In the case of N-first tracking of T-Ag, the loops would be expelled out the back of the C-terminal motor domains. N-first T-Ag hexamers track on ssDNA upon expulsion of the nontracking strand. Image credit: Nina Yao (Rockefeller University, New York, NY).

The eukaryotic CMG helicase, like SV40 T-Ag, is oriented head-to-head (i.e., N-to-N) at origins and it was long assumed to travel CTD tier-first for the same reasons as stated above for T-Ag (27). However, our studies on the eukaryotic CMG helicase clearly showed that CMG tracks NTD-tier first on DNA, and therefore the two CMGs are directed inward toward one another at an origin (18). We have also demonstrated that the head-to-head orientation of the two CMGs enables them to rip, or shear, the duplex apart, similar to the model of Fig. 6*B* and proposed here for T-Ag (40, 57).

It has been suggested in the eukaryotic field that a single CMG helicase that encircles dsDNA may be sufficient to unwind DNA at an origin (47). However, there is thus far no way to assemble only one CMG at an origin, a prerequisite to test this proposal. By contrast, the SV40 system enables a test of this model, because loading of only one T-Ag hexamer at an origin is made possible by mutation of critical features of either half of the SV40 origin as demonstrated (8, 61, 62). If a single T-Ag hexamer can unwind dsDNA, it should enable in vitro unwinding and replication using these mutant “half-origin” plasmids. However, numerous publications indicate that a single T-Ag hexamer is insufficient to melt the origin (8, 61, 62). These data support the proposal advanced here that two helicases translocating against one another are critical for the internal unwinding of dsDNA at an origin required for replication initiation as illustrated in Fig. 6*B* (see also Fig. 5).

### Revision of the “Rabbit Ear” DNA Loops during SV40 Replication.

Early EM studies documented DNA loops at the origin of SV40 during the initial stage of replication, often referred to as “rabbit ears” (19). The long-held view that T-Antigen tracks C-first implied that the two T-Ag hexamers move apart but initially remain connected to one another to produce “rabbit ear” DNA loops that protrude from between the two hexamers or from side ports in the hexamers (16, 17). A model of C-first movement of T-Ag double hexamers that remain connected in order to form “rabbit ear” DNA loops during initial origin unwinding is illustrated in Fig. 6*A* (see also Fig. 1*A* of ref. 15).

The early EM work that documented the “rabbit ear” DNA loops (19) used rotary shadowing and therefore lacked the resolution needed to determine, where the DNA loops emanated from the T-Ag double hexamer. However, the work shown here has implemented a cryo-EM “DNA pointer” strategy that has sufficient resolution to show that T-Ag tracks N-first, given that it tracks 3′–5′ as determined by previous studies (29, 30). We previously used this DNA pointer strategy with CMG to reveal that it tracked on DNA NTD-tier-first, and this conclusion is also supported by high-resolution cryo-EM studies by us and other labs for both CMG and archaeal Mcm (18, 47, 49). Given the new N-first directionality of SV40 T-antigen from the current study, the SV40 T-Ag joins all other eukaryotic replicative hexameric helicases in tracking N-first along ssDNA in the 3′–5′ direction. Therefore, the DNA loops would be produced by the two T-Antigen hexamers translocating toward one another while tracking 3′–5′ on opposite strands, thereby shearing the duplex between them apart to form the “rabbit ear’ loops, as they are ejected from the C-faces of the double hexamer as illustrated in Fig. 5*B*.

### Forces Required for Internal Unwinding of Duplex DNA.

The DNA shearing model in Fig. 6*B* requires that: 1) each T-Ag actively translocates while surrounding duplex DNA and 2) tracks on only the 3′–5′ strand of the duplex. Evidence for both criteria is clearly demonstrated herein (Figs. 3 and 4). This model also allows for the possibility that, while the rabbit ears are forming, replication can in principle proceed by leading strand synthesis alone. Interestingly, the location of priming and synthesis of each strand on either side of the origin was previously determined, but it was interpreted as lagging strand synthesis because of the presumption that the helicases moved C-first (63). With the N-first model we reveal here, these initial origin primed sites are likely primers for leading strand synthesis, not lagging strands.

Assuming two head-to-head helicase motors that each track on opposite strands, one can predict a force of 65pN is required to melt the DNA between them. This intelligence comes from single-molecule studies in which lambda DNA is attached to two optical traps on the two 3′ ends of the DNA (64). We have verified this is the case using RPA to report on ssDNA (65). Hence, if the two T-Ag helicases tracked on each opposing strand at 32.5 pN, the two opposite directed T-Ag helicases should satisfy the 65 pN required to melt the duplex. In fact, there is precedent for single hexameric and pentameric motors to produce forces that are far greater. A pentameric phage packaging motor produces up to 57-pN force (66), and the homohexameric *E. coli* FtsK partitioning motors pull with up to 60 pN (67). Thus, the forces required for two hexamer motors to achieve a total of 65 pN for dsDNA melting are well within reason.

### How is the Nontracked Strand Expelled from the Center of the Helicase?.

In the original “rabbit ears” experiments, most (~¾) of the replicating DNA molecules that were analyzed under normal conditions formed replication bubbles rather than rabbit ears (19). Furthermore, using a single-molecule approach where both ends of an SV40 origin-containing linear DNA were tethered, two divergent replication forks were observed (15). Together, these data argue that T-Ag does not operate exclusively as a double hexamer. Given that the two T-Ag hexamers are loaded head-to-head around dsDNA, this implies that one of the strands must be expelled from the interior of each hexamer in order for the two helicases to pass one another and establish divergent replication forks. Thus, T-Ag helicase encircles only one strand at a replication fork and excludes the other, an unwinding process referred to as steric exclusion. The eukaryotic CMG is also known to function in the steric exclusion mode (68).

How do these hexameric helicases transition from encircling dsDNA to encircling only one strand? We propose this transition is a natural consequence of two hexameric helicases that head into one another, and thus must pass each another to generate bidirectional replication forks. Initially, if inward directed helicases unwind DNA but do not pass one another, they disrupt dsDNA between them to form ssDNA strand and will produce ssDNA loops as they progress, with the ssDNA being extruded from the C-tier region (Fig. 6*B*). Keep in mind that the nontracking strand of one hexamer is the tracking strand of the other hexamer. Thus, only when the nontracking strands are expelled from the helicases can they pass one another, leaving a bubble of two separated ssDNA strands in their wake for leading and lagging strand synthesis as illustrated in Fig. 6*B*. In this event, the DNA shearing model quickly eliminates base pairing within the T-Ag central channels, and if the T-Ag does have a breathable interface, the conversion from double hexamer to single hexamer should be straightforward, as described below.

The mechanism of strand expulsion may be simple. It is well established that the hexameric helicases SV40 T-Ag, eukaryotic CMG, and *E. coli* DnaB have dynamic interfaces that spontaneously open and close transiently. The ability of the hexameric T-Ag ring to spontaneously open and close was previously demonstrated by the ability of T-Ag to unwind a stem-loop fork substrate with no free 3′ end for T-Ag loading (56). T-Antigen has also been shown to unwind ssDNA oligos annealed to an ssDNA circle, implying that T-Ag encircled the circular ssDNA (30, 58). Self-loading of CMG onto internal sections of ssDNA has been demonstrated by biochemical and single-molecule approaches, respectively (26, 65). The *E. coli* DnaB hexamer has also been directly shown to self-load onto a circular ssDNA (69). These observations reveal that closed hexamer rings “breathe” to provide transient gaps between protomers for DNA strand passage into the central chamber of the hexamer. Likewise, we propose that the non-tracking strand could exit the central chamber of these helicases through these same breathable interfaces.

### Generality of Head-to-Head Helicases at Origins.

The detailed biochemical and structural mechanics of how duplex DNA unwinding is initiated at origins has not been extensively studied in any system, but it is interesting to note that bacteria, eukaryotes, and archaea utilize head-to-head helicases that pass one another at origins to form bidirectional replication forks (70). This first step in replication initiation requires the melting of a significant amount of dsDNA, and it must be unwound by many turns in order to produce sufficient ssDNA that each large hexameric helicase can encircle only one strand to yield the classic steric exclusion helicase/replisome model. As an estimate for the eukaryotic system, given the 120 Å thickness of CMG, about four turns of duplex DNA must be unwound to accommodate one CMG on a ssDNA, and twice this amount of unwinding to accommodate two CMGs on ssDNA at origins for bidirectional unwinding. Thus far, only about 0.7 turns per helicase that contain three disrupted base pairs per CMG have been observed under static conditions (37, 71), and hence much more unwinding is required for origin initiation. We propose that the head-to-head DNA shearing model of Fig. 6*B* will produce the amount of ssDNA needed for unwinding dsDNA and for priming in the eukaryotic system (40), and this report indicates that this mechanism generalizes to the classic SV40 system.

The key elements of the T-Ag origin recognition and unwinding system are conserved in a group of mobile DNA elements first identified in *Staphylococcus aureus* known as SaPI’s. The structure of the SaPI origins mirrors that of SV40, with repeat sequences arranged in opposite orientations that recruit and direct the self-loading of two head-to-head homo-hexameric replicative helicases (72–74). Hence, besides the use of double hexamers to initiate dsDNA unwinding in eukaryotes and archaea, it may also occur in some bacterial settings. While the details of origin unwinding have yet to be fully explored in these systems, the structural organization of the origin and the self-loading of two oppositely oriented hexameric helicases strongly suggests that the DNA shearing mechanism described here is widely used in nature.

## Materials and Methods

### Reagents and Proteins.

Radioactive nucleotides were from Perkin Elmer. ATP was from Cytiva and adenylyl imidodiphosphate (AMP-PNP) was from Roche. DNA modification enzymes were from New England Biolabs. DNA oligonucleotides were from Integrated DNA Technologies except for those with MeP linkages, which were from Gene Link (Elmsford, NY). Full-length T-Antigen was from Millipore Sigma (SRP2093), and the N-terminal truncation, T-Ag^131–627^, of T-Ag was expressed in *E. coli* as detailed in the *SI Appendix*. Protein concentrations were determined using the Bio-Rad Bradford Protein stain using BSA as a standard.

### DNA Unwinding Assays.

Helicase assays made use of synthetic oligonucleotides configured as illustrated in the figures of this report. Reactions were performed at 37°C and typically contained, unless otherwise indicated, 40 nM T-Ag (as hexamer) and 0.5 nM radiolabeled DNA substrate in 20 mM Tris Acetate pH 7.6, 5 mM DTT, 0.1 mM EDTA, 10 mM MgSO_4_, 50 mM KCl, and 40 μg/ml BSA. T-Ag was preincubated at 37°C with the DNA for 10 min in the presence of 0.2 mM AMP-PNP, and reactions were started by the addition of 5 mM ATP along with 20 nM unlabeled trap DNA(s) to prevent reannealing of unwound product(s).

### EM Imaging and 3D Processing.

Cryo-EM analysis was performed on T-Ag^131–627^ either in the absence of DNA or bound to ssDNA 20-mers that were tipped with biotin-SA at either the 3′ or 5′ end. T-Ag was first incubated with the DNA, then SA was added before application to a cryo-EM grid, as detailed in the *SI Appendix*. Samples were then placed onto C-flat 2/1 holey carbon grids using a Thermo Fisher Vitrobot IV. Grids were analyzed using a Talos Arctica electron microscope, and images were captured using a Gatan K2 Summit direct electron detector. 2D averages and 3D reconstructions were performed as detailed in the *SI Appendix*.

Further details of the methods used in this report can be found in the *SI Appendix*.

## Supplementary Material

Appendix 01 (PDF)

## Data Availability

The cryo-EM 3D map of the J-domain truncated T-Ag hexamer (131-627 aa) in apo form at 5.6-Å resolution has been deposited in the Electron Microscopy Data Bank under accession code EMDB-28195.
