# Supplementary material for: SV40 T-antigen uses a DNA shearing mechanism to initiate origin unwinding
Source: Proc Natl Acad Sci U S A. 2022 Nov 28;119(49):e2216240119. doi: 10.1073/pnas.2216240119 (PMC9894130; doi:10.1073/pnas.2216240119)
Supplement: Supplementary file 1 — Appendix 01 (PDF) [file pnas.2216240119.sapp.pdf]

Supplementary Information Appendix for:

SV40 T-Antigen uses a DNA shearing mechanism to initiate origin unwinding

Lance D. Langston[1,2], Zuanning Yuan[3], Roxana Georgescu[1,2], Huilin Li[3*], and Michael E. O'Donnell[1,2,*]

1. DNA Replication Laboratory, The Rockefeller University, New York, United States
2. Howard Hughes Medical Institute, The Rockefeller University, New York, United States
3. Department of Structural Biology, Van Andel Institute, Grand Rapids, United States

This Appendix includes:
Detailed Experimental Procedures
2 Supplementary Figures
1 Table
References for Supplementary Material

Detailed Experimental Procedures

Purification of truncated T-Antigen (T-Ag$^{131-627}$): The gene encoding a truncated form of T-antigen (T-Ag$^{131-627}$) was synthesized (Biomatik) and inserted into pGEX-6P-1 (GeneScript) for E. coli expression. E. coli BL21(DE3) cells (Agilent Technologies) were transformed with the pGEX- T-Ag$^{131-627}$ plasmid and transformants were used to inoculate 100 ml LB media containing 100 μg/ml Amp and allowed to grow for 4 h at 37 °C with shaking. Then, 1 ml of the culture was used to inoculate each of 12 2L fluted flasks containing 1L LB + 100 μg/ml Ampicillin and allowed to grow at 29 °C with shaking. When the OD_{600} reached 0.66, each flask was chilled to 15 °C by manual shaking in an ice water bath, and then IPTG was added to 1mM final and the flasks were placed into a shaking incubator equilibrated at 15 °C. Cells were harvested after overnight expression by centrifugation at 5000 rpm in a Thermo Fisher RC3BP centrifuge and H6000A rotor for 30 min at 4 °C. Cells were resuspended in 50 ml GST Buffer (50 mM Tris pH 7.5, 500 mM NaCl,1 mM DTT, 1 mM EDTA, 10% glycerol) plus 2 ml of *E. coli* protease inhibitors (Sigma). Cells were lysed using a continuous flow pressure cell (Avestin C50) at 4 °C and cell debris was removed by centrifugation in a Thermo Fisher Lynx 6000 Rotor F14-6-250 at 12,500 rpm for 1 hour at 4 °C. The supernatant (212 ml, 4.2 g protein) was batch absorbed onto 3 ml of Glutathione Sepharose 4B (GE Healthcare) for 1h at 4 °C. Then the resin was recovered by centrifugation using a H6000A rotor at 1250 rpm for 9 min at 4 °C. Then we performed four "wash steps" as follows: beads were resuspended in 230 ml of GST buffer followed by collecting the beads by centrifugation as described above. This "wash step" was repeated an additional 3 times. Washed beads were then resuspended in 20 ml GST buffer and loaded into a XK16/20 column (GE Healthcare). The column was then connected to an ÄKTA FPLC system (GE Healthcare) and washed with 6 ml 50 mM Tris pH 8.0, 300 mM NaCl, 1 mM DTT, 1 mM EDTA, 10% glycerol. The protein was then eluted using GST elution buffer (50 mM Tris pH 8.0, 300 mM NaCl, 1 mM DTT, 1 mM EDTA, 10% glycerol, and 48 mM reduced L-Glutathione (Sigma) that was pre-adjusted to pH 8.0 using NaOH). The flow rate for elution was 0.5 ml/min using 23 ml elution buffer and collecting 0.5 ml fractions. Fractions were analyzed by 8% SDS PAGE and fractions containing the peak of T-Ag$^{131-627}$ were pooled and dialyzed against buffer A (20 mM Hepes pH 7.5, 50 mM NaCl, 1 mM DTT, 1 mM EDTA, 10% glycerol). The protein was then loaded onto a 2 ml SP Sepharose column (GE Healthcare) using an ÄKTA FPLC at a flow rate of 1 ml/min. The column was washed with 6 ml Buffer A. Protein was eluted using a 30 ml gradient from 50 mM to 1 M NaCl in buffer A at 0.5 ml/min collecting fractions of 1 ml. Fractions were evaluated by

8% SDS PAGE, and fractions 4-8, containing >95% pure T-Ag$^{131-627}$ were combined. Then 1.6 mg of T-Ag$^{131-627}$ was treated with 50 U of Prescission protease (GE Healthcare) at 4 °C overnight. To remove the cleaved GST domain, the protein preparation was passed over a GST column pre-equilibrated in 50 mM Tris pH 8.0, 10% glycerol, 300 mM NaCl, 1 mM DTT, 1 mM EDTA. T-Ag$^{131-627}$ is compared with full length T-Ag in an SDS PAGE gel in **SI Appendix Figure S2.**

DNA substrates: For all radiolabeled oligonucleotides, 10 pmol of oligonucleotide was labeled at the 5′ terminus with 0.05 mCi [γ-^{32}P]-ATP using T4 Polynucleotide Kinase in a 25 μl reaction for 30′ at 37°C according to the manufacturer's instructions. The kinase was heat inactivated for 20' at 80°C.

The substrate in Fig. 3 was made by mixing 2.4 pmol radiolabeled "C1 flush duplex 30" and 5 pmol unlabeled "Tailed Duplex 50" with 3.8 pmol unlabeled "Template 80 3′ tail". NaCl was added to a final concentration of 200 mM, and the mixture was heated to 90°C and then cooled to room temperature over a time frame of >1h. See ***SI Appendix*** **Table I** for oligo sequences.

The substrates in Fig. 4 were made by mixing 2.4 pmol radiolabeled "FD3" and 5 pmol unlabeled "FD2" with 3.8 pmol unlabeled "FD1". NaCl was added to a final concentration of 200 mM, and the mixture was heated to 90 °C and then cooled to room temperature over a time frame of >1 h. For lanes 7-12, FD2-MeP was used instead of FD2; for lanes 13-18 FD1-MeP was used in place of FD1. See ***SI Appendix*** **Table I** for oligo sequences.

The substrates in Fig. 5 were made by annealing unlabeled "Bottom 3′ tail" to radiolabeled "Top 3′ tail" (lanes 1-5); unlabeled "Bottom no tail" to radiolabeled "Top 3′ tail" (lanes 6-10); and unlabeled "Top no tail" to radiolabeled "Bottom 3′ tail" (lanes 11-15). 4 pmol of the radiolabeled strand was mixed with 6 pmol of unlabeled complementary strand, NaCl was added to a final concentration of 200 mM, and the mixture was heated to 90 °C and then cooled to room temperature over a time frame of > 1 h. See ***SI Appendix*** **Table I** for oligo sequences.

Sample preparations for cryo-EM analysis of T-Ag polarity on ssDNA

LT-Ag$^{131-627}$ was freshly dialyzed in 20 mM Tris-Acetate, pH 7.5, 40 mM potassium glutamate. Then, 8 μL of 1.85 pmol/μL of T-Ag$^{131-627}$ was combined with 1 μL of 15 pmol/μL 20-mer dT ssDNA-biotin templates (i.e., biotin labeled at either the 3′ or 5′ end) and incubated 20 minutes at room temperature. For both 3′ and 5′ biotin tagged DNA templates, we used either no nucleotide present, or + 1 mM AMP-PNP / 8mM MgAcetate. Then, 1 μL of 20 pmol/μL of SA tetramer (Streptavidin) was added and the mixture was incubated for an additional 5 minutes. The samples were flash frozen in liquid nitrogen for storage before thawing and application to EM grids.

Cryo-EM grid preparation and data collection. We pipetted 2.5 μl SV-40 T-Ag$^{131-627}$-ssDNA-SA samples onto C-flat 2/1 holey carbon grids that had previously undergone glow-discharge treatment. Using a Thermo Fisher Vitrobot IV, EM grids were then incubated in 100% humidity at 6 °C for 5 s before being blotted for 3 s and plunged into liquid ethane. Grids were put into a Talos Arctica electron microscope, and images were automatically captured in low-dose mode at 200 kV, 120,000x magnification, corresponding to 1.16 Å per pixel at the detector. Images were captured using a Gatan K2 Summit direct electron detector in super-resolution mode with an under-defocus ranging from 1.5 to 2.5 μm. The dose rate was 10 electrons per Å^2 per second, and the total exposure time was 6 s. The total dose was divided into 30-frame movies and each frame was exposed for 0.2 s.

EM Image processing and 3D reconstruction. We collected 1,652 raw cryo-EM movie micrographs of the SV40 T-Ag$^{131-627}$ apo form (no DNA), 1036 raw movie micrographs of SV40 T-Ag$^{131-627}$ with 5′ biotin-labeled Orient1 single-strand DNA, and 920 raw movie micrographs of SV40 T-Ag$^{131-627}$ with 3′ biotin-

labeled Orient2 ssDNA. The movie frames were first aligned and superimposed by the program Motioncorr2 (1). Using CTFFIND4 (2), the contrast transfer function parameters of each aligned micrograph were determined. Relion-3.1 (3) was used to complete the remaining processes, which included particle auto selection, 2D classification, 3D classification, 3D refinement, and density map post-processing. For the SV40-T-Ag apo dataset, automatic particle selection was performed, leading to 95,956 particle images. We then inspected the selected particles and sorted the particles by similarity to the 2D references. The bottom 10% of particles with the lowest Z-scores were removed from the particle pool. A total of 45,275 particles were used for further 3D classification. We derived five 3D models from the dataset and chose the best model for the final refinement. The other four models were distorted, and those particles were discarded. The final dataset had 22,375 particle images. These were used for further 3D refinement with C6 symmetry, resulting in a 5.8-Å EM map. The map resolution was estimated by the gold-standard Fourier shell correlation at the correlation threshold of 0.143. The density map was sharpened by applying a negative B-factor of -276 $Å^2$. We rigid body docked the published crystal structures of SV40 T-Ag AAA+-ZBD hexamer (PDB ID 1SVM) and the OBD (PDB ID 2FUF) into the EM map in ChimeraX (4). Similar to processing the apo SV40-Large T-Ag data, we used the SV40-Large T-Ag apo form template for particle picking of the SV40-Large T-Ag with 5' and 3' Bio-streptavidin labeled single-strand DNA complexes. After 2D classification, we generated 2D averages of these two complexes with various orientations.

DNA unwinding assays: Unless otherwise noted, all reactions (55 µl) were performed at 37 °C and contained 40 nM T-Antigen (as hexamer) and 0.5 nM radiolabeled DNA substrate in a buffer consisting of 20 mM Tris Acetate pH 7.6, 5 mM DTT, 0.1 mM EDTA, 10 mM $MgSO_4$, 50 mM KCl, and 40 µg/ml BSA. T-Antigen was pre-incubated at 37 °C with the DNA substrate for 10' in the presence of 0.2 mM AMP-PNP and the reactions were started by the addition of 5 mM ATP along with 20 nM unlabeled trap DNA(s) to prevent re-annealing of the unwound product(s).

At the times indicated in the figures, reaction aliquots (10 µl) were stopped by addition of 4 µl of buffer containing 150 mM EDTA/7% SDS. 1 µl Proteinase K was added and the mixture was incubated 10' at 37° C after which 3 µl STOP/LOAD buffer (0.1M EDTA, 5% SDS, 25% glycerol, and 0.01% each of xylene cyanol and bromophenol blue) was added and the sample was flash frozen in liquid nitrogen. Upon completion of the experiment, flash frozen reaction products were thawed quickly at 30 °C and separated on 10% or 15% native PAGE mini gels by electrophoresis at 100V in TBE buffer. Gels were washed in distilled water, mounted on Whatman 3MM paper, wrapped in plastic and exposed to a phosphor screen that was scanned on a Typhoon 9400 laser imager (GE Healthcare). Scanned gels were analyzed using ImageQuant TL v2005 software.

The trap oligos for Fig. 3 are unlabeled "C1 Flush Duplex 30" and "Duplex 50 COMP", which binds to Tailed Duplex 50 and prevents it from reannealing. The trap oligos for Fig. 4 are unlabeled FD3 and a pre-formed duplex fork structure consisting of "Tailed Duplex 50" annealed to "Duplex 50 COMP". The fork trap is present to prevent additional loading of T-Ag after the reaction is started. The trap oligos for Fig. 5 are "Bottom 5' tail" for the substrates where the top strand is radiolabeled and "Top 5' Tail" for the substrate where the bottom strand is radiolabeled. Oligo sequences are shown in ***SI Appendix*** **Table I**.

SUPPLEMENTARY FIGURES

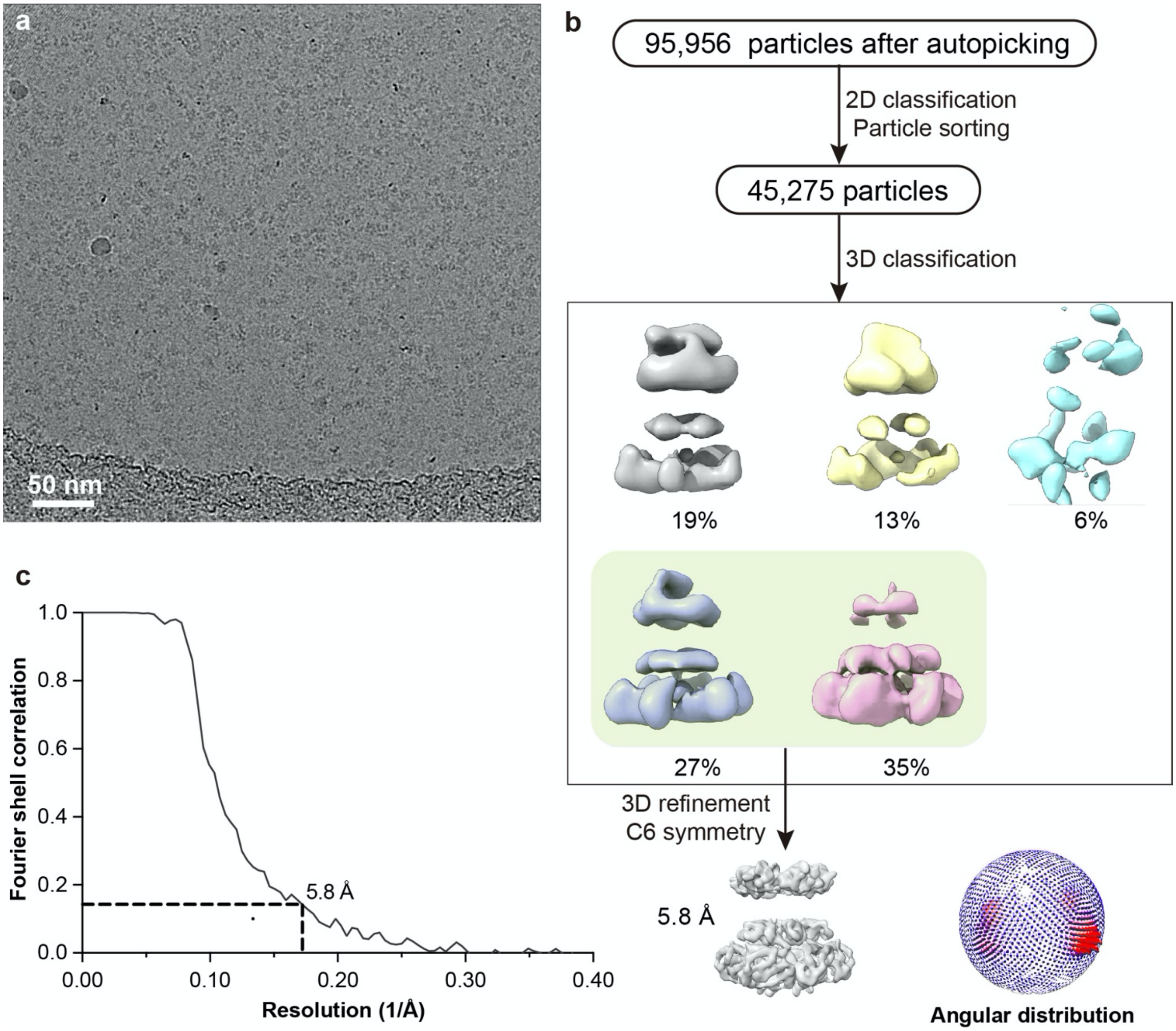

SI Appendix Figure S1. Cryo-EM 3D reconstruction of the purified T-Ag$^{131\text{-}627}$ in apo form. a) A representative raw micrograph of SV40-LT particles. **b)** 3D classification and refinement leading to the final 5.8-Å EM map. **c)** Gold-standard Fourier shell correlation. Inserted in the lower right corner of panel c is an angular distribution plot of particles used in the final reconstruction.

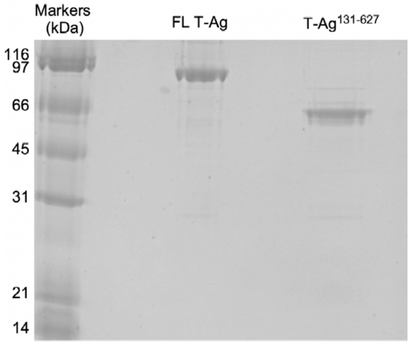

SI Appendix Figure S2. 12% SDS PAGE of SV40 large T-antigen and the N/C-terminal truncated version of T-Antigen (T-Ag$^{131\text{-}627}$) used in this report.

SI Appendix Table I

Oligo Name	Oligo Sequence (5'-3)	Modifications
Template 80 3' tail	GAGACCGAACGATCCTGTAATGTCCTAGCAAGCCAGAATTCGGCAGC GTCGCGATCTGCAGCCTTGCCAGAAATCTAGTGTTTTTTTTTTTTTTT TTTTTTTTTTTTTTTTTTTTTTTTTTT	
C1 Flush Duplex 30	CACTAGATTTCTGGCAAGGCTGCAGATCGC	
Tailed Duplex 50	TTTTTTTTTTTTTTTTTTTTTTTTTTTTTTTTTTTTTTTTTGACGCTG CCGAATTCTGGCTTGCTAGGACATTACAGGATCGTTCGGTCTC	
Duplex 50 COMP	GAGACCGAACGATCCTGTAATGTCCTAGCAAGCCAGAATTCGGCAGC GTCTTTTTTTTTTTTTTTTTTTTTTTTTTTTTTTTTTT*T*T*T*T*T	* = phosphorothioate linkages between the bases
FD1	GATCCTGTAATGTCCTAGCAAGCCAGAATTCGGCAGCGTCGCGATCT GCAGCCTTGCCAGAAATCTAGTGTTTTTTTTTTTTTTT*T*T*T*T*T	* = phosphorothioate linkages between the bases
FD2	CACTAGATTTCTGGCAAGGCTGCAGATCGCGACGCTGCCG	
FD3	TTTTTTTTTTTTTTTTTTTTTTTTTTTTTTTTTTTTTTTTTAATTCTG GCTTGCTAGGACATTACAGGATC	
FD1-MeP	GATCCTGTAATGTCCTAGCAAGCCAGAATT[mp-dC][mp-dG][mp-dG][mp-dC][mp-dA][mp-dG][mp-dC][mp-dG][mp-dT][mp-dC][mp-dG][mp-dC][mp-dG][mp-dA][mp-dT][mp-dC][mp-dT][mp-dG][mp-dC][mp-dA] GCCTTGCCAGAAATCTAGTGTTTTTT TTTTTTTT*T*T*T*T*T	mp represents methylphosphonate linkages between the bases<br><br>* = phosphorothioate linkages between the bases
FD2-MeP	CACTAGATTTCTGGCAAGGC[mp-dT][mp-dG][mp-dC][mp-dA][mp-dG][mp-dA][mp-dT][mp-dC][mp-dG][mp-dC][mp-dG][mp-dA][mp-dC][mp-dG][mp-dC][mp-dT][mp-dG][mp-dC][mp-dC][mp-dG]	mp respresents methylphosphonate linkages between the bases
Top 3' tail	GAAATAGGTTATTACTGAGTAGTATTTATTTAAGTATTGTTTGTGC ACTTGCCTGCAGGCCTTTTGAAAAGCAAGCATAAAAGATCTAAACA TAAAATCTGTAAAATAACAAGATGTAAAGATAATGCTAAATCATTT GGCTTTTTGATTTTTTTTTTTTTTTTTTTTTTTTTTTTTTTTTTTT TTTTT	
Top 5' tail	TTTTTTTTTTTTTTTTTTTTTTTTTTTTTTGAAATAGGTTATTACTG AGTAGTATTTATTTAAGTATTGTTTGTGCACTTGCCTGCAGGCCTTT TGAAAAGCAAGCATAAAAGATCTAAACATAAAATCTGTAAAATAAC AAGATGTAAAGATAATGCTAAATCATTTGGCTTTTTGATT	
Top no tail	GAAATAGGTTATTACTGAGTAGTATTTATTTAAGTATTGTTTGTGC ACTTGCCTGCAGGCCTTTTGAAAAGCAAGCATAAAAGATCTAAACA TAAAATCTGTAAAATAACAAGATGTAAAGATAATGCTAAATCATTT GGCTTTTTGATT	
Bottom 3' tail	AATCAAAAAGCCAAATGATTTAGCATTATCTTTACATCTTGTTATT TTACAGATTTTATGTTTAGATCTTTTATGCTTGCTTTTCAAAAGGCC TGCAGGCAAGTGCACAAACAATACTTAAATAAATACTACTCAGTAA TAACCTATTTCTTTTTTTTTTTTTTTTTTTTTTTTTTTTTTTTTT TTTT	
Bottom 5' tail	TTTTTTTTTTTTTTTTTTTTTTTTTTTTTTAATCAAAAAGCCAAATG ATTTAGCATTATCTTTACATCTTGTTATTTTACAGATTTTATGTTTA GATCTTTTATGCTTGCTTTTCAAAAGGCCTGCAGGCAAGTGCACAAA CAATACTTAAATAAATACTACTCAGTAATAACCTATTTC	
Bottom no tail	AATCAAAAAGCCAAATGATTTAGCATTATCTTTACATCTTGTTATT TTACAGATTTTATGTTTAGATCTTTTATGCTTGCTTTTCAAAAGGCC TGCAGGCAAGTGCACAAACAATACTTAAATAAATACTACTCAGTAA TAACCTATTTC	
Orient1	biotin-TTTTTTTTTTTTTTTTTTTT	5'-biotinylated dT_{20}
Orient2	TTTTTTTTTTTTTTTTTTTT-biotin	3'-biotinylated dT_{20}

REFERENCES FOR SUPPLEMENTARY APPENDIX

1. S. Q. Zheng *et al.*, MotionCor2: anisotropic correction of beam-induced motion for improved cryo-electron microscopy. *Nature Methods* **14**, 331-332 (2017).

2. A. Rohou, N. Grigorieff, CTFFIND4: Fast and accurate defocus estimation from electron micrographs. *Journal of Structural Biology* **192**, 216-221 (2015).

3. J. Zivanov *et al.*, New tools for automated high-resolution cryo-EM structure determination in RELION-3. *eLife* **7**, e42166 (2018).

4. T. D. Goddard *et al.*, UCSF ChimeraX: Meeting modern challenges in visualization and analysis. *Protein Science* **27**, 14-25 (2018).
